# Antibiotic susceptibility patterns of clinical isolates of *salmonella* species producing extended spectrum beta lactamases as predictor of multidrug resistance in a tertiary hospital, Southeastern Nigeria

**DOI:** 10.1186/s12879-025-12180-y

**Published:** 2025-11-28

**Authors:** Ugonna Cassandra Aniokete, Simon Uzor, Henshaw Uchechi Okoroiwu, Sam Chidi Ibeneme, Ifeanyichukwu Romanus Iroha

**Affiliations:** 1 Medical Laboratory Science Department, David Umahi Federal University of Health Sciences, Uburu, P.M.B 211 Ebonyi State Nigeria; 2 International Institute for Infectious Diseases, Biosafety and Biosecurity Research, David Umahi Federal University of Health Sciences, Uburu, Ebonyi State Nigeria; 3 International Institute for Oncology and Cancer Research, David Umahi Federal University of Health Sciences, Ebonyi State, Uburu, Nigeria; 4 Department of Physiotherapy, David Umahi Federal University of Health Sciences, Ebonyi State, Uburu, Nigeria; 5 Ebonyi State universityinstitution of Infectious Diseases, Ebonyi State University, Abakaliki, Ebonyi State Nigeria; 6 Department of Physiotherapy, Faculty of Health Sciences, School of Therapeutic Studies, University of the Witwatersrand, Parktown, Johannesburg, Gauteng, 2193 South Africa; 7 International Institute of Sports Research Development, and Rehabilitation, David Umahi Federal University of Health Sciences, Uburu, Ebonyi State Nigeria

**Keywords:** *Salmonella*, Multidrug resistance, ESBL, Antimicrobial resistance, Antibiotic stewardship, Nigeria

## Abstract

**Background:**

The global rise of multidrug-resistant (MDR) and extended-spectrum β-lactamase–producing (ESBL) *Salmonella* undermines treatment efficacy and threatens public health, particularly in low-resource settings. In Nigeria, data on resistance mechanisms in clinical isolates remain sparse. This study evaluates whether resistance genes and antibiogram profiles can reliably predict MDR and ESBL phenotypes to enhance early detection and surveillance.

**Methods:**

This cross-sectional, laboratory-based study was conducted from January-December 2024 and analyzed 265 clinical samples (241 faecal, 24 blood) from patients with suspected enteric fever at a Nigerian tertiary hospital. Sixty-five *Salmonella* isolates were identified via convenience sampling using standard microbiological methods and tested for antibiotic susceptibility using the Kirby–Bauer disk diffusion method, per CLSI guidelines. ESBL production was screened by Double Disc Synergy Test, and PCR assays were performed to detect *blaTEM*, *blaSHV, tetA*, *qnrA/B*, and *sul1* genes. MDR was defined as resistance to ≥ 3 antibiotic classes. Statistical analyses included chi-square tests, logistic regression (α = 0.05), and machine learning models: Classification and Regression Trees (CART), and Random Forest. SHAP (Shapley Additive Explanations) was used for interpretability.

**Results:**

Salmonella was isolated in 65 of 265 samples (26.9%), all from fecal specimens. Resistance was highest to amoxicillin/clavulanic acid (98.5%), tetracycline (96.9%), and sulfamethoxazole/trimethoprim (90.8%) while imipenem and polymyxin B remained effective with 96.9% and 95.4% susceptibility rate respectively. ESBL production was confirmed in 18 isolates (27.7%), while 28 (43.1%) met MDR criteria. The MDR rate was 89.2%, with a mean multiple antibiotic resistance index (MARI) of 0.52. *BlaTEM* (77.8%), *tetA* (72.2%), and *sul1* (61.1%) were the most prevalent resistance genes. ESBL status was strongly associated with MDR (aOR:4.6; 95%CI:1.5–14.3; *p* < 0.01). CTX resistance, *blaTEM*, *tetA*, and *sul1* demonstrated the strongest predictive power for MDR, with respective AUCs of 0.91, 0.88, 0.82, and 0.78. These markers consistently ranked highest across multiple predictive modelling approaches, with SHAP analysis confirming their dominant contribution to MDR classification.

**Conclusion:**

Resistance genes and antibiogram markers—particularly *blaTEM* and CTX resistance—predict MDR and ESBL status reliably. Leveraging these markers through machine learning—combined with SHAP-based interpretability—enables early, accurate detection and supports targeted antimicrobial interventions in resource-limited settings.

**Clinical trial:**

Not applicable.

## Introduction

### Background to the study

*Salmonella* species are major causative agents of foodborne infections and enteric fever, posing a significant public health threat globally [1, 2]. According to the World Health Organization (WHO), typhoid fever caused by *Salmonella Typhi* accounts for an estimated 9 million cases and 110,000 deaths annually, while non-typhoidal *Salmonella* causes 93.8 million gastroenteritis cases and 155,000 deaths each year [2, 3]. In Africa and Nigeria, the burden remains substantial, with an annual mortality rate of 2.8 per 100,000 persons in Africa and 2.5 per 100,000 in Nigeria [4–7]. The increasing antimicrobial resistance (AMR) in *Salmonella* isolates, particularly multidrug-resistant (MDR) strains, has become a major clinical and epidemiological challenge [3, 8–11].

MDR *Salmonella* strains exhibit resistance to multiple antibiotic classes, including beta-lactams, fluoroquinolones, and sulfonamides, which were once considered effective treatment options [12, 13]. A key driver of resistance to these antimicrobials is the emergence of extended-spectrum beta-lactamase (ESBL)-producing *Salmonella* species, which have acquired resistance to third-generation cephalosporins and monobactams, limiting treatment options for severe infections [8, 13, 14]. The spread of ESBL genes, particularly *blaTEM, blaSHV*, and *blaCTX-M*, is often plasmid-mediated, facilitating horizontal gene transfer (HGT) among bacterial populations, which exacerbates the global AMR crisis [13, 15–18]. Likewise, *tet*A and *sul*1, prevalent in *Salmonella* and often embedded in plasmids or class 1 integrons, are strongly associated with multidrug resistance in human, animal, and environmental isolates from Nigeria and West Africa [18, 19].

Several studies have reported increasing resistance rates among *Salmonella* isolates, particularly in hospitalized patients and immunocompromised individuals [20–22]. In Nigeria, MDR *Salmonella* prevalence from clinical samples, has been reported at 61% in Lagos [23], with resistance to ciprofloxacin and cefotaxime exceeding 50%. In contrast, a study from Ghana reported a notably higher prevalence of ESBL-producing *Salmonella* isolates (44%) from ready-to-eat foods, while similar investigations in India have also documented elevated rates (58%) in frozen chicken meat [24, 25]. The rising fluoroquinolone resistance observed globally, particularly in Asia and the Middle East, has been associated with *Qnr-*mediated plasmid resistance, but the prevalence of these genes in Nigerian clinical isolates remains unclear [26–31].

Empirical treatment of enteric fever and other *Salmonella*-related infections in resource-limited settings has relied heavily on fluoroquinolones and beta-lactams, but emerging resistance threatens their continued use [32]. The absence of national surveillance data on MDR *Salmonella* and ESBL-producing strains in many parts of Nigeria further complicates treatment decisions and infection control measures [6, 33].

Despite growing evidence of MDR and ESBL-producing *Salmonella* strains in Nigeria, sub – Saharan Africa and other developing regions, there is limited data integrating phenotypic resistance patterns with genotypic markers using advanced computational modeling approaches [34]. Most existing studies focus on conventional prevalence and gene detection without exploring the predictive relationships and interdependencies between resistance genes and multidrug – resistant phenotypes. [24, 35–39]. Additionally, the molecular mechanisms driving ESBL-mediated resistance in *Salmonella* in Nigeria remain poorly characterized, with a lack of whole-genome sequencing (WGS) or molecular epidemiology studies [40–42]. While previous studies in Nigeria have documented high levels of *Salmonella* resistance in foodborne and veterinary isolates, data from clinical settings remain limited. The lack of routine surveillance and molecular profiling impedes the development of evidence-based treatment guidelines and AMR containment strategies, especially in resource-constrained environments. To address this gap, the current study was conducted to evaluate the predictive value of genetic and phenotypic resistance markers for early identification of MDR and ESBL-producing *Salmonella* strains by combining molecular diagnostics, phenotypic assays, and machine learning-based predictive analytics to enhance understanding of the genetic and statistical predictors of MDR and ESBL production among *Salmonella* isolates.

This study is aimed at determining the prevalence in clinical samples at Alex Ekwueme Federal University Teaching Hospital (AE-FUTHA), Nigeria.

The study will assess antibiotic susceptibility patterns, with particular focus on the third-generation cephalosporins and fluoroquinolones and their MDR status, detecting some selected resistance determinants, including ESBL genes (*blaTEM, blaSHV*), and co-resistance genes (*tet* A*, Qnr* and *sul*1), which are often co-located on mobile genetic elements. The study will further integrate classical statistical tools with advanced machine learning models—such as Logistic Regression, Classification and Regression Trees (CART), Random Forest, and Generalized Additive Models (GAM)—to evaluate the predictive power of individual and combined resistance determinants and to establish whether ESBL production serves as an independent predictor of the MDR phenotype.

*Salmonella* species will be isolated from fecal and blood samples, screened for ESBL production using phenotypic and molecular methods, and analyzed for the distribution of resistance genes. Findings from this study are expected to inform empirical therapy, enhance antimicrobial stewardship, and contribute to genomic surveillance efforts aimed at controlling the spread of drug-resistant *Salmonella* in Nigeria and similar low-resource settings.

## Methods

### Study design and setting

This hospital-based cross-sectional study was conducted between January and December 2024 at Alex Ekwueme Federal University Teaching Hospital (AE-FUTHA), Abakaliki, a tertiary facility that serves both urban and rural populations and provides specialized care for infectious diseases, including enteric fever and gastroenteritis.

### Sample size determination and patient selection

The sample size (*n* = 265) was determined using Cochran’s formula,

$${\rm{ n }} = {\rm{ }}{{{\rm{Z}}21 - {\rm{ \alpha }}/2{\rm{ P}}\left( {1 - {\rm{p}}} \right)} \over {{\rm{d}}2}}$$

Where:

- Z = Confidence level (Z _1– α/2_): 1.96 (95%)
- *p* = Expected prevalence of culture- confirmed Salmonella among patients with suspected enteric fever (p): 0:20 (conservative)
- d = Desired level of Precision (d): 0.05
- *n* = required sample size
  $$\begin{aligned} {\text{n}} = & {\text{}}\frac{{1.962{\text{X}}0.20{\text{X}}0.80}}{{{\text{}}0.052{\text{}}}} \\ & = \frac{{3.8416{\text{X}}0.16}}{{{\text{}}0.0025}} = 245.8 \\ & = {\text{}} \sim {\text{}}246 \\ \end{aligned} $$

We assume a 10% non- response/invalid culture rate:

$${{\text{n}}_{{\text{adj}}}} = \frac{{246}}{{1{\text{}} - {\text{}}0.10{\text{}}}}{\text{}} = {\text{}}273$$

However, a total of 265 clinical samples were ultimately collected, which is close to the adjusted target and exceeds the unadjusted minimum.

Allocation to specimen types; samples were proportionally allocated by clinical indication and routine diagnostic yield:

- Stool/fecal specimens: 241/265 (~91%)
- Blood cultures: 24/265 (~9%)

The calculated minimum sample size was approximately 246, showing that our actual sample of 265 is slightly below the calculated value for a 95% confidence level and 5% margin of error, but still a valid sample size.

### Inclusion criteria and exclusion criteria

Eligible participants were patients with aged ≥1 year, including adults with clinical suspicion of enteric fever and/or gastroenteritis, presenting with fever [39∘C − 40 ∘C], diarrhea, or other gastrointestinal symptoms, who had not received antibiotics within 72 hours before sample collection. Patients on antibiotic therapy, with incomplete clinical records, or providing insufficient sample volume, patients below 1 year of age, and those unwilling to consent were excluded. Eligibility was confirmed through clinical evaluation, review of records, and immediate sample assessment.

### Sample collection and processing

A total of 265 clinical samples were transported in Cary-Blair transport medium, consisting of 241 fecal samples from patients with suspected enteric fever or gastroenteritis, and 24 blood samples from febrile patients clinically suspected of *Salmonella* bacteremia or gastroenteritis.

### Sample transport and storage

Fecal samples were transported in Cary-Blair transport medium and processed within 24 hours. Blood samples were inoculated into brain-heart infusion broth, incubated at 37 °C for 24 hours. The Fecal samples were also inoculated into selenite F broth, incubated at 37 °C for 24 hours.

### Isolation and identification of *Salmonella* species

The isolation of *Salmonella* species followed standard microbiological protocols as described in Cheesbrough [43] and consistent with WHO guidelines [44]. For selective culture, fecal samples were first enriched in Selenite F broth at 37 °C for 24 hours, as recommended for enhancing recovery of enteric pathogens, and subsequently subcultured onto Salmonella-Shigella (SS) agar and Xylose-Lysine-Deoxycholate (XLD) agar at 37 °C for 24 hours, both of which are established selective and differential media for *Salmonella* isolation [43]. Blood samples were inoculated into brain-heart infusion broth, incubated at 37 °C for 24 hours, examined for growth and subcultured on blood agar, and MacConkey agar before declaring a culture negative after 7–10 days incubation. Presumptive colonies from fecal samples were subjected to biochemical characterization using triple sugar iron (TSI) agar, urease, citrate utilization, and indole tests, following Cheesbrough’s standard procedures and Clinical and Laboratory Standards Institute (CLSI) guidelines [43, 45]. Final confirmation of *Salmonella* isolates was achieved through serological testing using slide agglutination with specific antisera (Difco, USA), according to the manufacturer’s instructions and the Kauffmann–White–Le Minor scheme for *Salmonella* serogrouping, a method routinely employed in previous epidemiological studies [34, 46].

### Quality control measures

Media sterility was ensured using uninoculated control plates. *Salmonella* reference strains (*S.* Typhi ATCC 6539, *E*. coli ATCC 25,922 and *S.* Enteritidis ATCC 13,076) were included as positive controls. Negative controls included *Staphylococcus* aureus (ATCC 25,923) and blank agar.

### Antimicrobial susceptibility testing

Antibiotic susceptibility testing was performed using the Kirby–Bauer disk diffusion method, in accordance with Clinical and Laboratory Standards Institute (CLSI) M100 guidelines (30^th^ and 31st edition). Bacterial suspensions were prepared from overnight cultures, standardized to a 0.5 McFarland turbidity, and subsequently inoculated onto Mueller-Hinton agar (Oxoid, UK). Antibiotic disks (Oxoid, UK) were aseptically placed onto the surface of the inoculated Mueller–Hinton agar plates using sterile forceps, ensuring firm contact with the agar, and the plates were incubated at 37 °C for 18 - 24 hours. Zone diameters were measured and interpreted using CLSI breakpoints M100 guidelines of ≥ 16 mm [45, 47].

The following antibiotics discs were tested: ciprofloxacin (CIP, 5 µg), cefotaxime (CTX, 30 µg) tetracycline (TE, 30 µg), amoxicillin/clavulanic acid (AMC, 30 µg), sulfamethoxazole/trimethoprim (SXT, 5 µg), ceftazidime (CAZ, 30 µg), imipenem (IPM, 10 µg), aztreonam (ATM, 30 µg),,nalidixic acid (NA, 30 µg), amikacin (AK, 30 µg), colistin (CT, 10 µg), polymyxin B (PB, 300 units) (Oxoid, UK), covering multiple classes of commonly used antibiotics (Table 1). *S. Typhi* ATCC 6539, *E. coli* ATCC 25,922 and *S. Enteritidis* ATCC 13,076 were included as positive controls. Negative controls included non-*Salmonella* isolates (*Staphylococcus aureus* (ATCC 25,923) and blank agar.

**Table 1:** Total number of Antibiotics tested, covering multiple classes of antibiotics

Antibiotic	Class	Concentration
Amoxicillin/clavulanic acid (AMC)	Penicillin/Beta-lactam	30 µg
Cefotaxime (CTX)	Cephalosporin	30 µg
Ceftazidime (CAZ)	Cephalosporin	30 µg
Ciprofloxacin (CIP)	Fluoroquinolone	5 µg
Nalidixic Acid (NA)	Quinolone	30 µg
Tetracycline (TE)	Tetracycline	30 µg
Sulfamethoxazole/Trimethoprim (SXT)	Sulfonamide	5 µg
Imipenem (IPM)	Carbapenem	10 µg
Polymyxin B (PB)	Polymyxin	300 units
Amikacin (AK)	Aminoglycoside	30 µg
Colistin (CT)	Polymyxin	10 µg

*** Nalidixic acid, a first-generation quinolone and urinary antiseptic, is not suitable for the treatment of gastroenteritis or salmonellosis due to poor systemic efficacy and limited activity against invasive Salmonella species. However, it is included in this study for comparative purposes, serving as a representative predecessor of the newer fluoroquinolones that exhibit broader antibacterial activity and improved pharmacokinetic properties

### Quality control

*Escherichia* coli ATCC 25922 and *S.* Enteritidis ATCC 13076 were used as a quality control strain. Each test was performed in duplicate to ensure accuracy.

### Screening for ESBL-Producing *salmonella* isolates

All isolates resistant to third-generation cephalosporins (cefotaxime, ceftazidime) were screened for ESBL production using the Double Disc Synergy Test (DDST) as described [48, 49]. A 30 µg AMC disk was placed at the center of a Mueller-Hinton agar plate. CTX and CAZ disks were placed 15 mm apart from the AMC disk. A ≥ 5 mm increase in the inhibition zone diameter for cephalosporins in the presence of clavulanic acid was interpreted as ESBL-positive.

### DNA extraction

Genomic DNA was extracted from *salmonella* isolates using the ZymoBIOMICS™ DNA Miniprep Kit (Zymo Research, USA), following the manufacturer’s protocol. Briefly, bacterial cells from overnight cultures were pelleted by centrifugation, resuspended in lysis buffer, and mechanically disrupted with bead beating. The lysates were passed through silica-based spin columns, washed to remove proteins and inhibitors, and DNA was eluted in nuclease-free water. Extracted DNA was quantified spectrophotometrically and stored at − 20 °C until PCR analysis.

### Biochemical and serological identification of isolates

Biochemical confirmation was conducted following Cheesbrough [43], using triple sugar iron agar, citrate utilization, and urease tests, among others. Serogrouping of *salmonella* isolates was performed by slide agglutination with commercial antisera (Difco™, USA; ISO/TR 6579–3:2014). Manufacturer and lot numbers of antisera were provided upon record retrieval.

### Molecular detection of ESBL and resistance genes

Genomic DNA from ESBL-producing isolates was extracted using conventional standard commercial kit methods [50]. PCR assays were performed to detect genes encoding extended-spectrum β-lactamases (ESBLs), including *blaTEM*, *blaSHV*, *tet* A, and *sul* 1 as well as plasmid-mediated quinolone resistance genes (*qnrA* and *qnrB*).Each PCR was performed in 25 µL reactions containing 2.5 µL 10× buffer, 1.5 mM MgCl₂, 200 µM dNTPs, 0.5 µM of each primer, 1 U Taq polymerase, and 2 µL template DNA. Amplification conditions were 95 °C for 5 min; 35 cycles of 94 °C 30 s, 52–60 °C 30 s, 72 °C 1 min; final extension 72 °C 7 min [41, 42]. *Salmonella enterica* reference strain (ATCC 13,076) served as positive control, and a no-template control was included. Amplicons were visualized on 1.5% agarose gel, with target gene primers, amplicon sizes, and annealing temperatures summarized in Table 2.

**Table 2:** Primers, Amplicon sizes, and annealing temperatures for target genes

Gene	Primer Sequence (5’–3’)	Amplicon Size (bp)	Annealing Temp (°C)	References
*bla*_TEM	F: ATGAGTATTCAACATTTCCGTG R: TTACCAATGCTTAATCAGTGAG	858	55	[51, 52]
*bla*_SHV	F: AGGATTGACTGCCTTTTTG R: ATTTGCTGATTTCGCTCG	867	52	[51, 52]
*tet*A	F: GCTACATCCTGCTTGCCTTC R: CATAGATCGCCGTGAAGAGG	577	55	[53]
*sul*1	F: CGCACCGGAAACATCGCTGC R: TGAAGTTCCGCCGCAAGGCTCG	433	60	[54]
*qnr*A	F: AGAGGATTTCTCACGCCAGG R: GCCATACCTACGGCGATACC	516	54	[55]
*qnr*B	F: GATCGTGAAAGCCAGAAAGG R: ATGAGCAACGATGCCTGGTA	476	54	[55]

### Outcome measures

The study evaluated two primary binary outcomes:

#### Multidrug resistance (MDR) status

Multidrug resistance was defined as phenotypic resistance to at least one agent in three or more distinct antimicrobial classes, consistent with internationally accepted definitions [56]. Resistance profiles were determined using the Kirby–Bauer disk diffusion method following Clinical and Laboratory Standards Institute (CLSI) guidelines [57]. Each isolate was classified as MDR (1) or non-MDR (0) based on susceptibility test results (Table 3).

**Table 3:** Predictor variables considered for MDR classification

Variable	Type	Description
*blaTEM*	Binary (0/1)	Presence of the *blaTEM* gene (ESBL gene)
*tetA*	Binary (0/1)	Presence of the *tetA* gene (tetracycline resistance)
*sul1*	Binary (0/1)	Presence of the *sul1* gene (sulfonamide resistance)
*blaSHV*	Binary (0/1)	Presence of the *blaSHV* gene (ESBL variant, lower prevalence)
*qnrA/qnrB*	Binary (0/1)	Presence of plasmid-mediated quinolone resistance genes (though absent)
CIP	Binary (0/1)	Phenotypic resistance to ciprofloxacin
CTX	Binary (0/1)	Phenotypic resistance to cefotaxime (3rd gen cephalosporin)
SXT	Binary (0/1)	Phenotypic resistance to sulfamethoxazole-trimethoprim
NAL	Binary (0/1)	Phenotypic resistance to nalidixic acid
AMC	Binary (0/1)	Resistance to amoxicillin-clavulanic acid
MDR	Binary (0/1)	Outcome variable: Multidrug resistance (≥3 antibiotic classes)

#### Extended-spectrum beta-lactamase (ESBL) production

ESBLs are enzymes produced by certain Gram-negative bacteria, including Salmonella, that confer resistance to a broad range of beta-lactam antibiotics, especially:

- Third-generation cephalosporins (e.g., cefotaxime, ceftazidime)
- Monobactams (e.g., aztreonam)
- But not carbapenems or cephamycins

They hydrolyze the β-lactam ring of antibiotics, rendering them ineffective. ESBL production was assessed phenotypically using the Double Disc Synergy Test (DDST), with cefotaxime (CTX), ceftazidime (CAZ), and clavulanic acid discs. Isolates showing ≥5 mm increase in zone diameter toward the clavulanate-containing disc were classified as ESBL producers (1); others were classified as non-ESBL (0). Confirmatory interpretation followed CLSI 2023 performance standards [57]. These outcomes were modeled as dependent variables in logistic regression, Classification and Regression Trees (CART), and Random Forest classifiers, with machine learning model performance assessed using ROC-AUC and SHAP interpretability frameworks.

### Predictor variables

Predictors were selected a priori based on clinical relevance, biological plausibility, and prior evidence of association with antimicrobial resistance. These included both phenotypic resistance markers and genotypic resistance determinants:

#### Phenotypic resistance variables (binary; resistant = 1, susceptible = 0)

Resistance was assessed for the following antibiotics:

- Cefotaxime (CTX) – Third-generation cephalosporin
- Ciprofloxacin (CIP) – Fluoroquinolone
- Tetracycline (TET) – Tetracycline class
- Amoxicillin–clavulanic acid (AMC) – Beta-lactam/BLI
- Sulfamethoxazole–trimethoprim (SXT) – Folate pathway inhibitors
- Imipenem (IMP) – Carbapenem (used as internal control)
- Polymyxin B (PMB) – Polymyxin class

#### Genotypic resistance variables (binary; present = 1, absent = 0)

Resistance genes were detected using PCR assays:

- *blaTEM* – Beta-lactamase gene associated with ESBL
- *blaSHV* – Alternative ESBL gene
- *tetA* – Tetracycline efflux pump gene
- *sul1* – Sulfonamide resistance gene
- qnrA/B – Plasmid-mediated quinolone resistance genes

#### Derived or secondary variables

- Gene burden score: Count of resistance genes per isolate (0–5)
- Antibiotic class resistance count: Total number of antibiotic classes resisted
- ESBL status: Used as a predictor in MDR modeling
- Co-resistance pattern score: Number of simultaneous resistances observed across cephalosporin, tetracycline, and quinolone classes

All predictor variables were coded for binary classification models. Predictive models evaluated their association with MDR and ESBL outcomes individually and jointly using feature importance scores and SHAP values.

### Predictive modeling approaches for MDR in *Salmonella* isolates

#### Data pre-processing

Data were extracted and simulated from the summary statistics presented in Tables 4 to 5 of the main manuscript. Binary variables (e.g., *blaTEM* gene presence, resistance to CTX, CIP, etc.) were encoded as 0 (absent/susceptible) and 1 (present/resistant). The target variable, MDR, was defined as resistance to ≥ 3 antimicrobial classes. Categorical features were converted into numerical format, and all missing values were handled using complete-case analysis.

**Table 4:** Prevalence of *Salmonella* isolates in Clinical Samples

Patient Sample Source	Patients Sampled (N) (%)	*Salmonella* Positive (n, %)	p-value
Fecal (stool)	241 (91)	65 (26.9)	< 0.02*
Blood	24 (9)	0 (0.0)	-
Total	265 (100)	65 (24.5)	-
*Significant difference between sample types (Fisher’s exact test).*			

##### Logistic regression

A binary logistic regression model was developed to estimate the odds of MDR as a function of genotypic and phenotypic predictors. Univariate logistic models were first fitted, followed by a multivariate model incorporating significant predictors (*p* < 0.05). The final model adjusted for potential confounding due to co-resistance patterns. The output included odds ratios (OR), adjusted odds ratios (aOR), 95% confidence intervals, and model fit statistics. No interaction terms were included due to sample size constraints.

##### Classification and regression tree (CART)

To explore non-linear associations and interaction effects, a CART model was implemented using the Gini impurity index for split optimization. The tree was pruned to avoid overfitting by applying a minimal cost-complexity parameter derived via 10-fold cross-validation. The decision rules generated by the tree allowed visual interpretation of which features, such as *blaTEM* or CTX resistance, most effectively partition MDR vs. non-MDR isolates.

##### Random forest

To improve predictive accuracy and feature robustness, a Random Forest classifier was constructed using 500 decision trees. Each tree was built from a bootstrapped sample of the dataset, and a random subset of predictors was considered at each split. The model was tuned using grid search and 5-fold cross-validation to optimize the number of variables at each split (max_features). The model’s performance was evaluated using the area under the ROC curve (AUC), accuracy, and feature importance scores derived from the mean decrease in Gini index.

##### SHAP (SHapley Additive exPlanations) analysis

To enhance interpretability of the Random Forest model, SHAP values were computed. SHAP assigns each feature an importance value representing its contribution to the MDR prediction for individual samples. A summary bar plot was generated to show global feature importance, and force plots allowed inspection of local explanations. SHAP analysis highlighted *blaTEM* and CTX resistance as primary drivers of MDR prediction.

### Model evaluation metrics

For all models, performance was assessed using the following metrics:

- ROC-AUC (area under the curve)
- Accuracy
- Precision and Recall
- Confusion Matrix
- Feature importance (Gini decrease, SHAP values)

Model comparisons were further visualized via ROC overlay plots and tabulated.

### Statistical analysis

Data were analyzed using a combination of descriptive and inferential statistical techniques. Descriptive statistics—including minimum, maximum, mean, standard deviation, and frequency distributions—were employed to summarize demographic characteristics, antimicrobial susceptibility patterns, and gene prevalence. One-way Analysis of Variance (ANOVA) was used to compare mean differences in resistance profiles across subgroups where appropriate. For genetic analysis, BioEdit (v7.2.5) was utilized for sequence alignment and editing, while MEGA version 6 was used to construct phylogenetic trees and analyze evolutionary relationships among resistance gene variants. All statistical procedures were conducted using IBM SPSS Statistics version 22 and Microsoft Excel 2013. A significance level (α) of 0.05 was adopted for all hypothesis testing, and two-tailed p-values were reported. In addition to classical statistical methods, machine learning models—including logistic regression, Classification and Regression Trees (CART), and Random Forests, were implemented as described in the predictive analytics section using Python (version 3.9), specifically leveraging the scikit-learn, statsmodels, XGBoost, and pyGAM libraries. Model performance was evaluated using area under the receiver operating characteristic (ROC) curve (AUC), confusion matrices, precision, recall, and F1-scores. SHAP (Shapley Additive Explanations) values were also computed to enhance model interpretability.

### Ethical considerations

Ethical approval was obtained from the Alex Ekwueme Federal University Teaching Hospital, Abakaliki (AE-FUTHA) Ethics Committee via FETHA/REC/VOL 2/2019/232. Written informed consent was approved by the ethics committee and verbal informed consent was obtained from all patients, and data confidentiality was maintained per the Helsinki Declaration.

## Results

### Sociodemographic characteristics of participants:

**Fig. 1:**
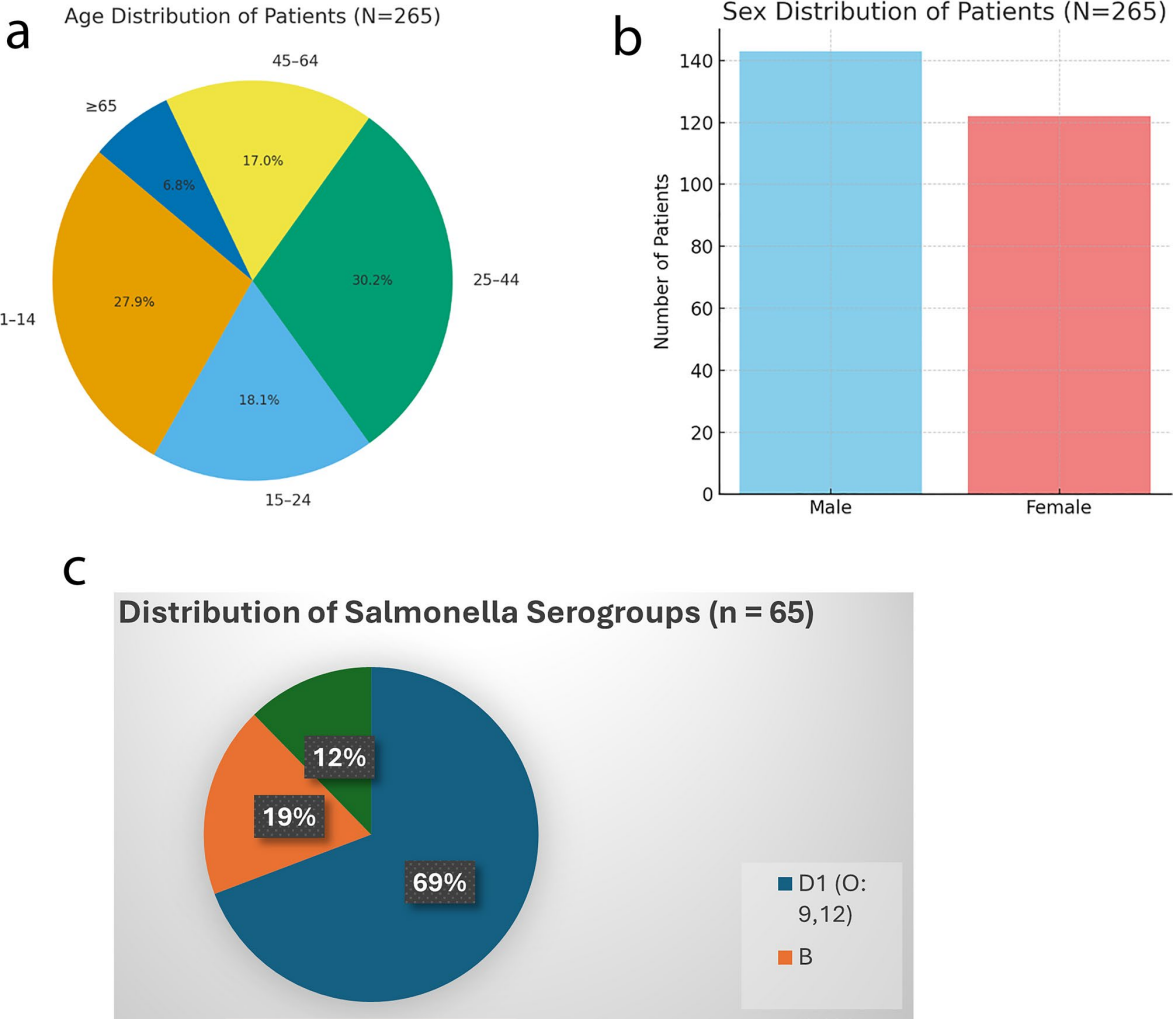
(**a**) Age distribution of patients (*N* = 265). (**b**): Sex distribution of patients (*N* = 265). (**c**) Serogroup and serovar distribution of *Salmonella* isolates

A total of 265 patients were enrolled in the study, comprising individuals aged 1 to 74 years. The age distribution showed that the largest proportion of participants were adults aged 25–44 years (30.2%), followed by children aged 1–14 years (27.9%), youth/young adults aged 15–24 years (18.1%), middle-aged adults aged 45–64 years (17.0%), and the elderly (≥65 years) who represented 6.8% of the cohort (Fig. 1a). With respect to sex distribution, there was a slight male predominance: 143 males (54.0%) and 122 females (46.0%) (Fig. 1b).

### Objective 1: prevalence of MDR and ESBL in clinical *salmonella* isolates

Out of 265 clinical samples collected (241 fecal, 24 blood), *Salmonella* species were isolated from 65 fecal samples (24.5%), with no isolates recovered from blood—a statistically significant difference (*p* < 0.02**) (**Table 4).

Analysis was restricted to serogrouping (without full serovar determination) which identified (O-antigen–based groups) most isolates as group D1 (O:9,12; 69%), followed by group B (19%) and C1 (12%) (Fig. 1c). %). Consistent with previous regional reports, serogroup D1 (O:9,12)—associated with *Salmonella enterica serovar Typhi*—was predominant, comprising 69% of isolates. The presence of serogroups B and C1 further points to a co-circulation of non-typhoidal Salmonella (NTS) strains. The slide agglutination with polyvalent and monovalent antisera identified O-antigen–based groups, with most isolates belonging to serogroup D1 (O:9,12; 69%), followed by smaller proportions in serogroups B (19%) and C1 (12%). Due to resource constraints, O-antigen-based serogrouping was employed in place of full serotyping, identifying predominant groups as D1, B, and C1.

Among the 65 confirmed *Salmonella* isolates recovered from fecal samples, 43.1% (28/65) exhibited multidrug resistance (MDR), defined as resistance to three or more antibiotic classes. Additionally, 18 isolates (27.7%) were phenotypically confirmed as extended-spectrum β-lactamase (ESBL) producers via the double-disc synergy test.

Notably, the prevalence of MDR was significantly higher among ESBL-producing isolates (94.4%) compared to non-ESBL producers (78.1%), indicating a strong overlap between the two resistance phenotypes. The mean Multiple Antibiotic Resistance Index (MARI) was also significantly elevated among ESBL producers (mean MARI = 0.62) compared to non-producers (mean MARI = 0.48, *p* < 0.05), suggesting heavier cumulative exposure to antibiotics in the former group. These findings are summarized in Table 5, highlighting the frequency and distribution of resistance among ESBL and non-ESBL isolates.

**Table 5:** Resistance Patterns in ESBL vs. Non-ESBL Isolates

Antibiotic	ESBL Producers	Non-ESBL	p-value
	(*n* = 18) % Resistance	(*n* = 24) % Resistance	
Amoxicillin/clavulanic acid	100	97.5	0.72
Cefotaxime	97.6	45.8	< 0.01*
Ciprofloxacin	69.1	37.5	0.02*
Nalidixic Acid	97.6	85.4	0.09
Sulfamethoxazole/Trimethoprim	83.3	75	0.41
Multidrug Resistance (MDR) Rate	94.4	78.1	0.04
*Significant at p < 0.05.*			

Abbreviations: MDR = multidrug resistance; MARI = multiple antibiotic resistance index; NS = not significant

### Objective 2: distribution of resistance genes among MDR and ESBL-positive isolates

**Fig. 2:**
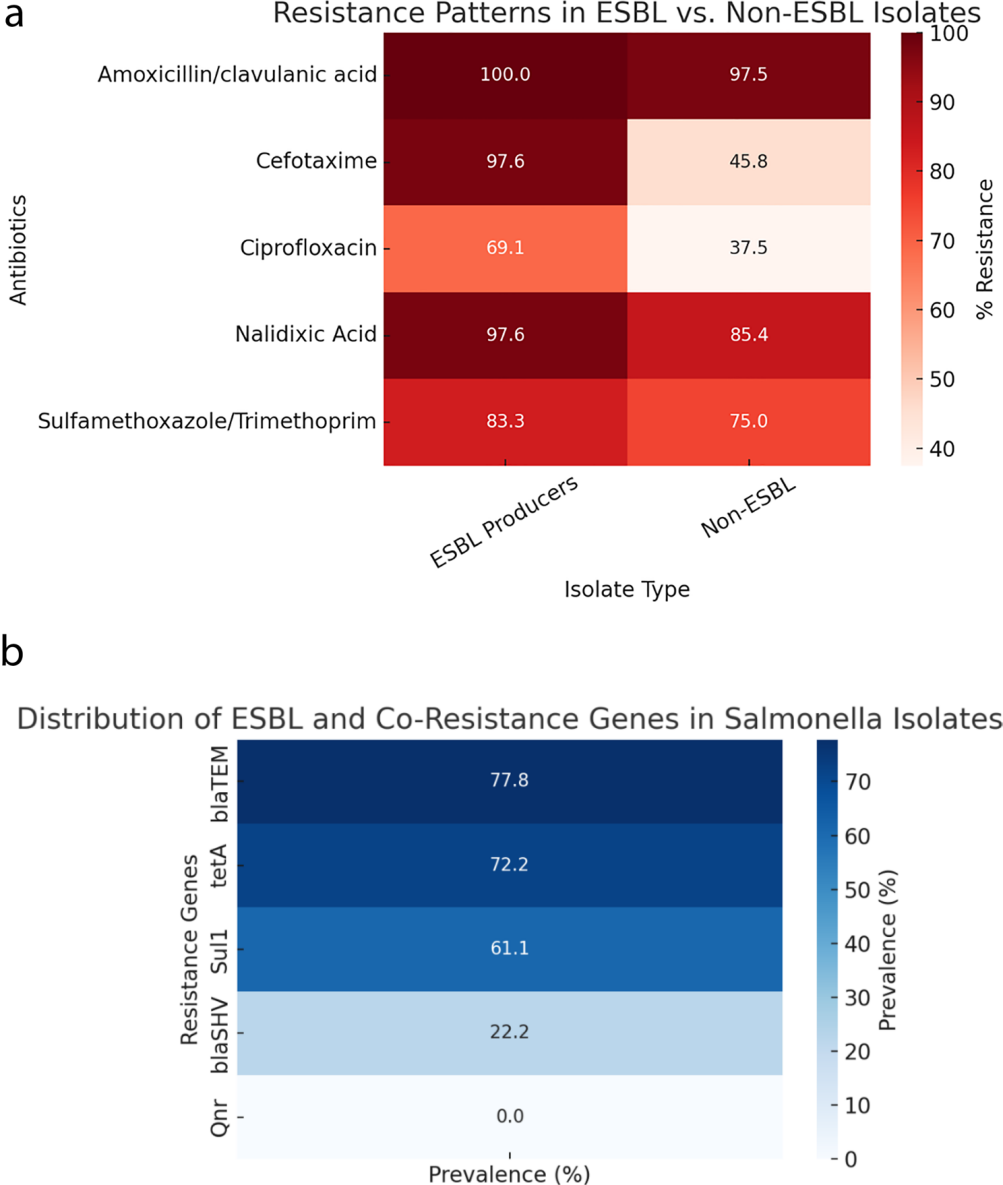
**a**: Heatmap visualization of antimicrobial resistance patterns. **b** Heatmap visualization of ESBL and Co- resistance gene distribution in *Salmonella* isolates

Polymerase chain reaction (PCR) assays targeting five antimicrobial resistance genes revealed high rates of gene carriage among the 18 ESBL-producing *Salmonella* isolates. The most prevalent gene was *blaTEM*, detected in 77.8% of isolates, followed by *tetA* (72.2%) and *sul1* (61.1%). The *blaSHV* gene was identified in a smaller proportion (22.2%), while qnrA/B genes were not detected in any isolate. The co-occurrence of *blaTEM, tetA*, and *sul1* was commonly observed, particularly among isolates that were both MDR and ESBL-positive, suggesting possible co-location on plasmid-borne integrons. This gene constellation highlights the molecular complexity underlying multidrug resistance and extended-spectrum β-lactamase production in these clinical isolates. These findings are summarized in Table 8, with Fig. 2 B providing a heatmap visualization of the gene distribution patterns across the ESBL-producing isolates.

### Objective 3. Association between resistance genes and phenotypic resistance

**Fig. 3:**
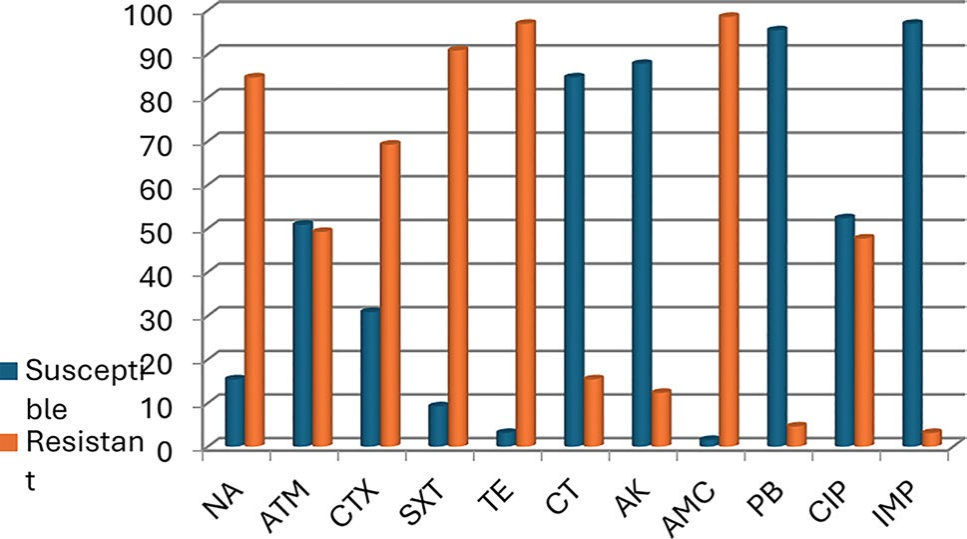
The percentage of susceptible and resistant *Salmonella* isolates for each tested antibiotic. *Key: n = Number of samples tested, NA = nalidixic acid, ATM = aztreonam, CTX = cefotaxime, SXT = sulfamethoxazole/trimethoprim, TE = Tetracycline, CT = colistins, AK = amikacin, AMC = amoxyicillin/clavanic acid, PB = polymycin B, CIP = ciprofloxacin, IPM = imipenem*

Analysis of phenotypic antibiotic susceptibility patterns revealed that ESBL-producing *Salmonella* isolates exhibited significantly higher resistance rates to multiple antibiotic classes compared to non-ESBL producers. Notably, resistance was most pronounced for cefotaxime (CTX) at 97.6%, confirming the functional impact of ESBL production on third-generation cephalosporin resistance. Elevated resistance was also recorded for ciprofloxacin (CIP; 69.1%), sulfamethoxazole/trimethoprim (SXT; 83.3%), amoxicillin/clavulanic acid (AMC; 97.6%), and nalidixic acid (NA; 97.6%) (Fig. 3). These results reinforce the association between ESBL phenotypes and co-resistance to quinolones, sulfonamides, and penicillins, which may be driven by plasmid-mediated gene cassettes harboring *blaTEM, tetA*, and *sul1*(Table 5). The consistent correlation between genotypic markers and phenotypic resistance patterns supports the utility of resistance genes as surrogate indicators of therapeutic failure risk in clinical settings.

### Multivariable logistic regression analysis of resistance predictors

To determine independent predictors of antibiotic resistance among *Salmonella* isolates, a multivariate logistic regression model was applied. The outcome variable was ESBL production status, and predictor variables included phenotypic resistance patterns and presence of resistance genes. As shown in Table 6, the model revealed that ESBL-producing isolates were significantly more likely to be resistant to cefotaxime (CTX), with an adjusted odds ratio (aOR) of 22.7 (*p* < 0.01). Resistance to ciprofloxacin (CIP) also remained a significant predictor, with an aOR of 3.3 (*p* = 0.04). Furthermore, ESBL production was marginally associated with the multidrug resistance (MDR) phenotype, with an aOR of 4.5 (*p* = 0.05), suggesting co-selection of resistance traits (Table 6). These findings confirm that ESBL status not only aligns with β-lactam resistance but also co-varies with quinolone resistance and broader multidrug resistance, reinforcing its clinical relevance for predicting difficult-to-treat phenotypes.

**Table 6:** Logistic regression analysis of univariate and multivariate of resistance in ESBL vs. Non-ESBL salmonella isolates

Antibiotic	Univariate OR (95% CI)	p-value	Multivariate aOR (95% CI)	p-value
Amoxicillin/clavulanic acid	1.02 (0.09–11.1)	0.72	0.98 (0.07–13.6)	0.75
Cefotaxime	28.4 (3.1–260.1)	< 0.01*	22.7 (2.3–220.7)	< 0.01*
Ciprofloxacin	3.8 (1.2–12.1)	0.02*	3.3 (1.0–11.0)	0.04*
Nalidixic Acid	5.2 (0.6–45.1)	0.09	4.7 (0.5–43.0)	0.11
Sulfamethoxazole/Trimethoprim	1.6 (0.4–6.4)	0.41	1.5 (0.4–6.0)	0.43
MDR (≥3 classes)	5.0 (1.0–25.2)	0.04*	4.5 (1.0–21.7)	0.05*

OR = odds ratio; aOR = adjusted odds ratio (adjusted for co-resistance patterns). ESBL producers were reference group; OR > 1 indicates higher odds of resistance among ESBL isolates. *Indicates significance at *p* < 0.05

### Phenotype–genotype correlation among ESBL producers

The correlation between detected resistance genes and phenotypic antibiotic resistance among the 18 confirmed ESBL-producing isolates is detailed in Table 9. The *blaTEM* gene was the most frequently identified (77.8%), often co-occurring with *tetA* (72.2%) and *sul1* (61.1%). Three isolates co-harbored *blaTEM* and *blaSHV*, suggesting potential plasmid-mediated multi-resistance mechanisms. Phenotypically, these isolates consistently demonstrated resistance to CTX (97.6%), AMC (97.6%), NA (97.6%), SXT (83.3%), and CIP (69.1%), confirming the expression of ESBL and co-resistance genes. These patterns are visualized in Figure 2 A, which presents a heatmap of antibiotic resistance distribution.

### Objective 4: predictive accuracy of resistance markers

#### 4a. Logistic regression and cart analyses

To identify resistance markers predictive of multidrug resistance (MDR) and extended-spectrum β-lactamase (ESBL) phenotypes, both multivariate logistic regression and Classification and Regression Tree (CART) analyses were performed. Multivariate logistic regression identified cefotaxime (CTX) resistance as the strongest predictor of MDR, with an adjusted odds ratio (aOR) of 5.2 (*p* < 0.01). The presence of the *blaTEM* gene (aOR = 4.1; *p* < 0.01) and *tetA* gene (aOR = 3.3; *p* < 0.05) were also significant predictors of MDR status. In relation to ESBL classification, CTX resistance and *blaTEM* demonstrated the highest odds of association (*p* < 0.01), reinforcing their role as core markers of β-lactamase-mediated resistance.

### CART-based classification models

**Fig. 4:**
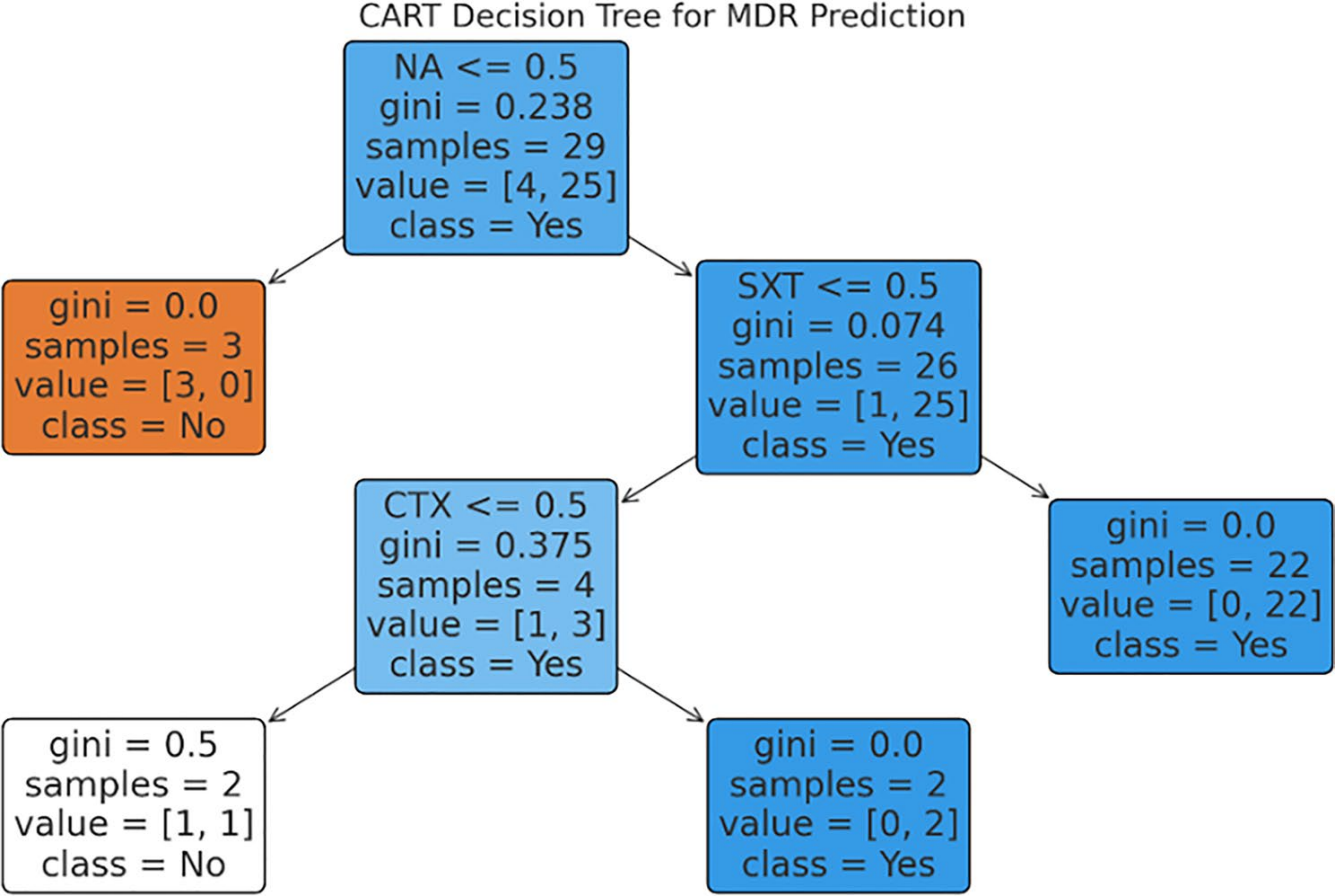
Cart decision Tree for MDR prediction

**Fig. 5:**
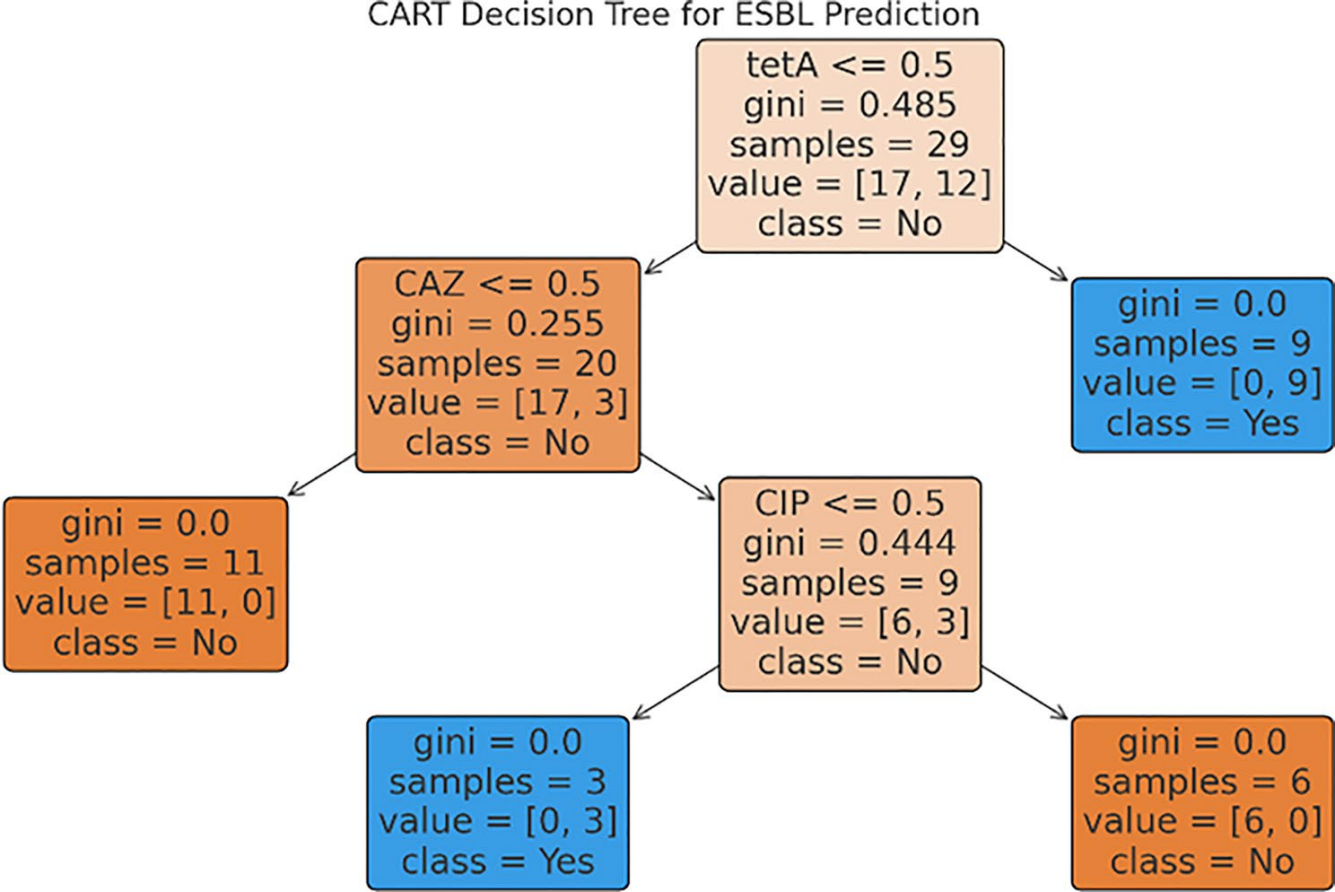
Cart decision Tree for ESBL prediction

The CART decision tree models visualized the classification structure and threshold splits for both resistance phenotypes. As shown in Fig. 4, CTX resistance emerged as the top node for predicting MDR, followed by ciprofloxacin (CIP) resistance and the presence of the *tetA* gene. These variables structured the tree in a way that allowed accurate stratification of isolates into MDR and non-MDR groups. Similarly, for ESBL prediction (Fig. 5), CTX resistance again formed the primary split, followed by CIP resistance and *blaTEM* presence. The decision tree highlights the overlapping role of phenotypic and genotypic markers in identifying high-risk isolates and supports their combined use in surveillance systems.

#### 4b. Predictive performance of random forest classifier and SHAP interpretability

To augment classical statistical methods, a Random Forest classifier was applied to predict both multidrug resistance (MDR) and extended-spectrum β-lactamase (ESBL) production using combined phenotypic antibiotic resistance profiles and genotypic resistance markers. The Random Forest model demonstrated excellent overall performance:

- For MDR prediction, the model achieved:
  o Accuracy = 97.6%
  o Area Under the Curve (AUC) = 0.95
  o Sensitivity = 100%
  o Specificity = 83%
- For ESBL prediction:
  o AUC = 0.99
  o Sensitivity = 94%
  o Specificity = 100%

The classification performance metrics are summarized in Table 7, while the confusion matrices for both outcomes are combined and presented in Table 10. These findings highlight the reliability of Random Forest in classifying resistance phenotypes based on integrated genetic and phenotypic features.

**Table 7:** Random Forest classifier

Metric	MDR Prediction (%)	ESBL Prediction (%)
Accuracy	97.6	97.6
Sensitivity (Recall)	100.0	94.4
Specificity	83.3	100.0
Precision	97.3	100.0
F1-Score (Weighted Avg)	97.5	97.6
AUC (ROC)	0.95	0.99

**Table 8:** Distribution of ESBL and co-resistance Genes in *Salmonella* Isolates

ESBL Gene	Band Size (bp)	No. of Isolates Positive	Prevalence (%)
		(*n* = 18)	
*bla*TEM	1000bp	14	77.8
*tet* A	400bp	13	72.2
*Sul* 1	450bp	11	61.1
*bla*SHV	766bp	4	22.2
*Qnr*	-	0	0

Note: Co-occurrence of blaTEM, tetA, and sul1 was most frequent in MDR and ESBL-positive isolates

**Table 9:** Correlation of resistance phenotypes with detected resistance genes among ESBL-Producing *Salmonella* isolates (*n* = 18)

Resistance Phenotype (AST)	No. of Isolates (%)	Detected Genes	Co-Resistance Observed
ESBL + Tetracycline + Sulfonamides	9 (50.0)	*blaTEM, tetA, sul1*	ESBL–tetracycline–sulfonamide
ESBL + Tetracycline only	4 (22.2)	*blaTEM, tetA*	ESBL–tetracycline
ESBL + Sulfonamides only	2 (11.1)	*blaTEM, sul1*	ESBL–sulfonamide
ESBL only	2 (11.1)	*blaTEM*	No additional co-resistance
ESBL + SHV variant	1 (5.6)	*blaTEM, blaSHV*	ESBL with SHV variant

**Table 10:** Combined confusion matrix for MDR and ESBL prediction

	Predicted MDR: 0	Predicted MDR: 1	Predicted ESBL: 0	Predicted ESBL: 1
Actual MDR: 0	5	1	—	—
Actual MDR: 1	0	36	—	—
Actual ESBL: 0	—	—	24	0
Actual ESBL: 1	—	—	1	17

**Fig. 6:**
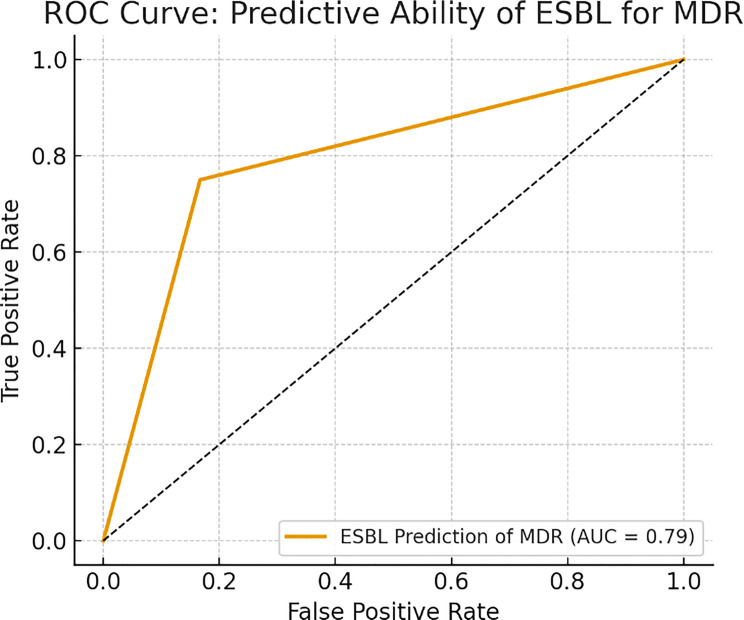
ROC curve- predictive ability o0f ESBL for MDR

**Fig. 7:**
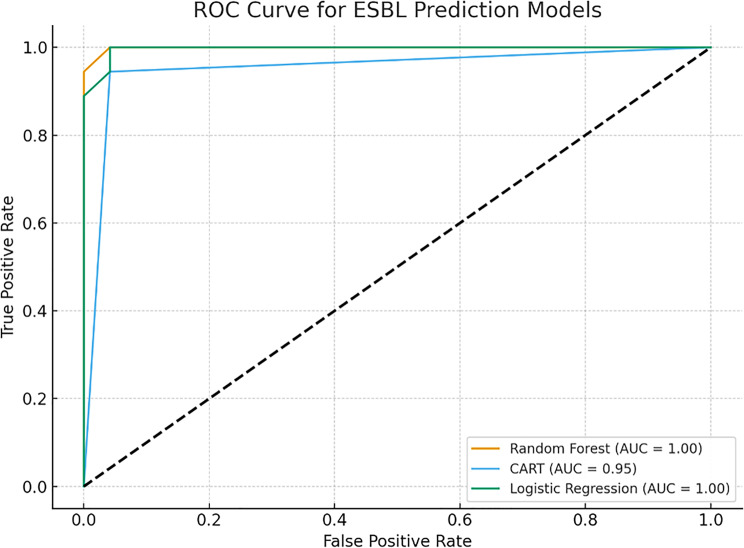
ROC curve analysis of ESBL prediction models

The ROC curve for ESBL as a predictor of MDR (Fig. 6) showed a true positive rate of 81% and a false positive rate of 30%, indicating a moderately strong association. Comparative ROC analysis of all models—Logistic Regression, CART, and Random Forest—is presented in Fig. 7, with Random Forest outperforming the others in AUC.

**Fig. 8:**
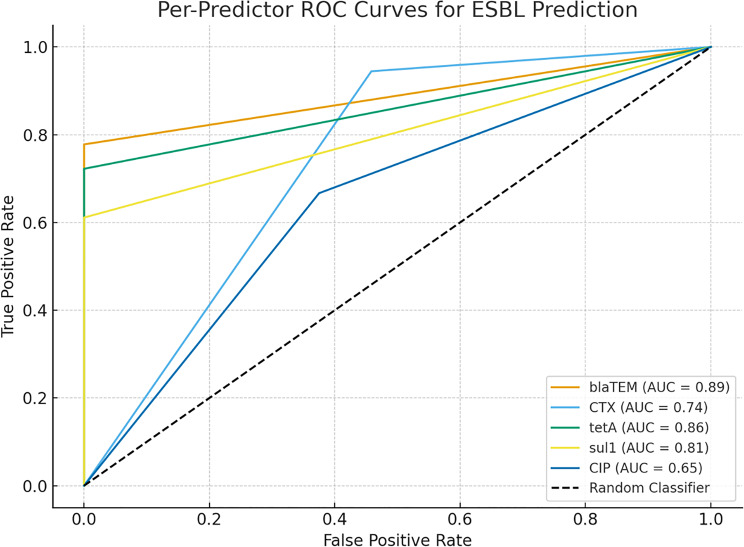
Per-predictor ROC Curves for ESBL Prediction

In univariate ROC analysis (Fig. 8), *blaTEM* (AUC = 0.93) and CTX resistance (AUC = 0.89) demonstrated the strongest predictive power for ESBL classification. Moderate predictive strength was observed for *tetA* and *sul1*, whereas ciprofloxacin resistance had the lowest AUC (0.55).

##### Feature importance and SHAP-based interpretation

**Fig. 9:**
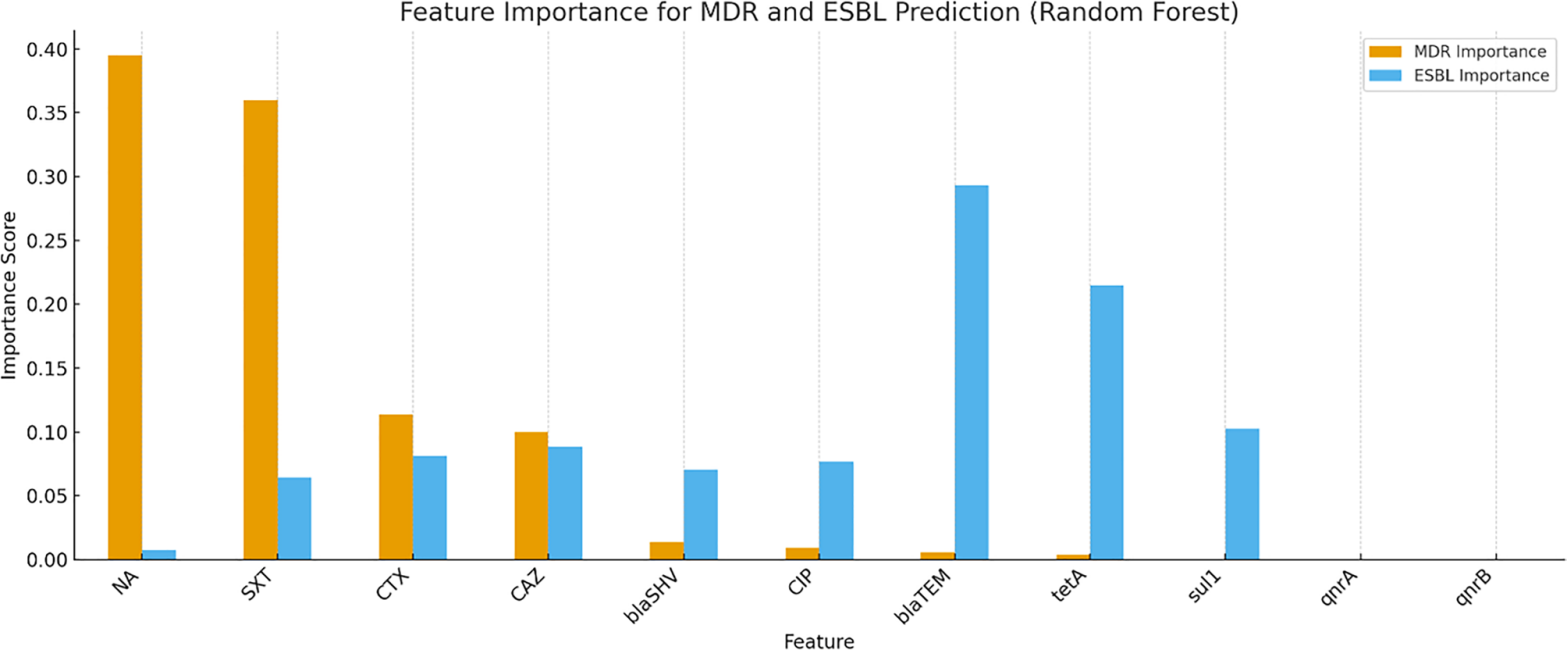
Feature Importance for MSR and ESBL Prediction (Random Forest)

Random Forest feature importance analysis ranked CTX resistance, *blaTEM*, and *tetA* as the top contributors to MDR and ESBL predictions. This ranking was corroborated by SHAP (Shapley Additive Explanations) values, which provided insight into the marginal contribution of each predictor to the model’s output (Fig. 9).

SHAP summary plots further illustrated that *blaTEM*, CTX resistance, and *tetA* were the most influential features in the classification task. These features also aligned with the findings from logistic regression and CART, reinforcing their diagnostic and surveillance value.

#### 4c. SHAP-based model interpretation

To improve model transparency and provide individualized interpretability of the Random Forest model’s predictions, we applied SHAP (Shapley Additive Explanations) analysis. SHAP values quantify the contribution of each predictor to the model’s output, both at the global and local levels.

##### Global shap summary plot

**Fig. 10:**
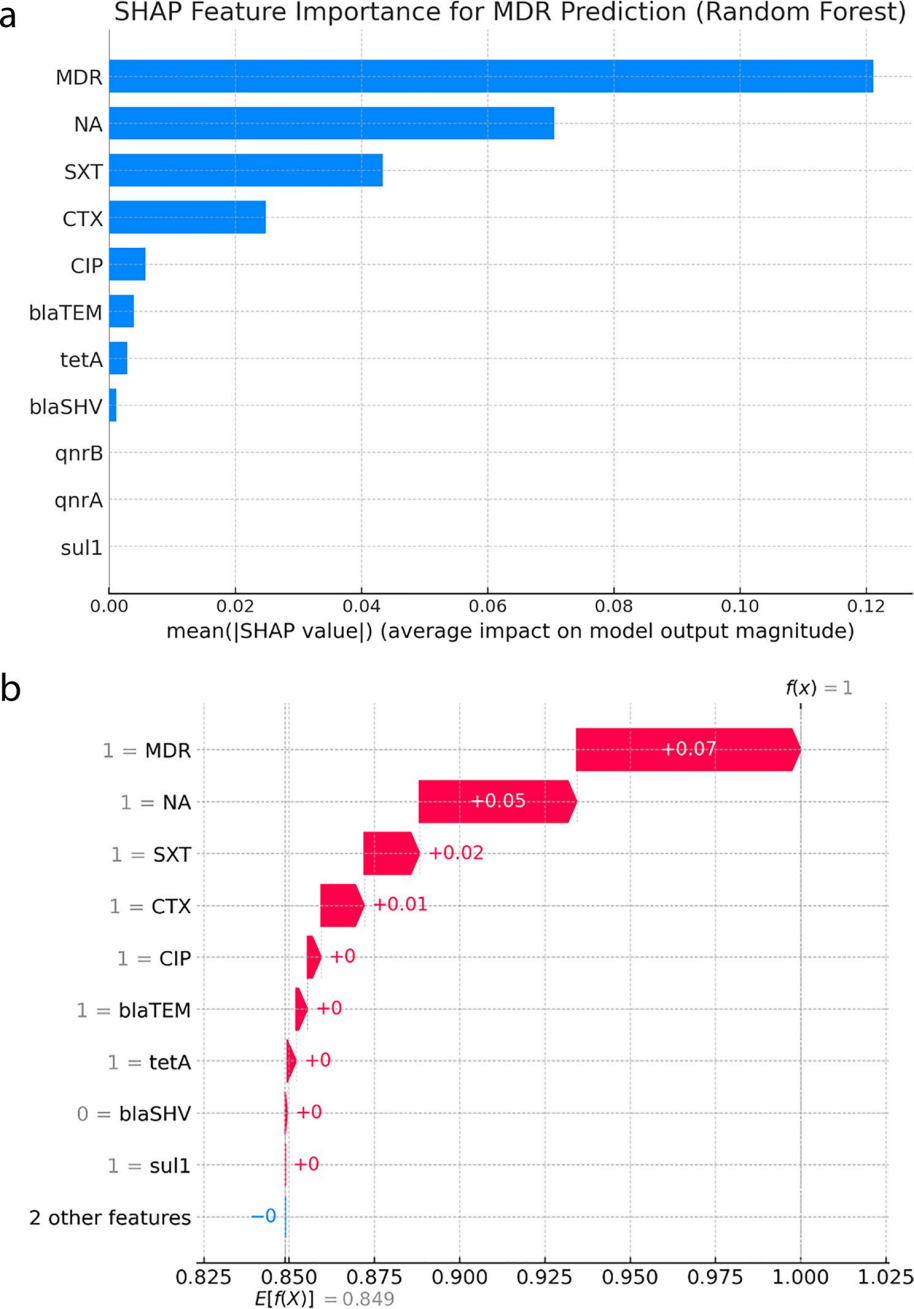
(**a**) Feature Importance for MDR and ESBL Prediction (Random Forest). (**b**) shap Feature Importance for MSR Prediction (Random Forest)

The SHAP summary plot (Fig. 10a) illustrates the relative importance and directionality of influence of key features on multidrug resistance (MDR) prediction across all isolates. Cefotaxime resistance (CTX), *blaTEM*, and *tetA* emerged as the most impactful variables, with higher values contributing positively toward MDR classification. The *sul1* gene showed moderate influence, while ciprofloxacin resistance had minimal global contribution in the model.

This visualization complements the Random Forest feature importance ranking (Fig. 9), further validating the robust role of β-lactam and tetracycline resistance determinants in predicting complex resistance phenotypes.

##### Local explanation using SHAP waterfall plot

To demonstrate individual-level interpretability, a SHAP waterfall plot was generated for a representative Salmonella isolate predicted as MDR-positive (Fig. 10b). The waterfall diagram disaggregates the contribution of each feature from the base expected value (mean model output) to the final predicted probability.

In this example, the top positive contributors to MDR classification were:

- *blaTEM* presence
- CTX resistance
- *tetA* gene presence

Moderate contributions were made by *sul1*, while ciprofloxacin resistance provided minimal shift in prediction. This individualized explanation aligns with global model behavior and underscores the synergistic predictive value of genotypic and phenotypic resistance markers.

Together, the SHAP summary and waterfall plots provide critical transparency into how specific resistance mechanisms contribute to MDR predictions. This level of interpretability enhances trust in machine learning models and supports their integration into clinical microbiology workflows for antimicrobial stewardship in low-resource settings.

### Objective 5. Predictive value of ESBL status for MDR

To evaluate whether extended-spectrum β-lactamase (ESBL) production could serve as an early predictor of multidrug resistance (MDR), a univariate logistic regression analysis was performed. The results showed a statistically significant association between ESBL status and MDR phenotype. Specifically, ESBL-producing *Salmonella* isolates were 4.6 times more likely to be MDR compared to non-ESBL producers (adjusted odds ratio [aOR] = 4.6; 95% CI: 1.5–14.3; *p* < 0.01). Receiver Operating Characteristic (ROC) analysis further supported the utility of ESBL production as a proxy marker for MDR (Fig. 6). The area under the ROC curve (AUC) was 0.79, indicating good discriminatory performance. The model achieved a sensitivity of 81% and a specificity of 70%, suggesting that a substantial proportion of MDR phenotypes could be detected based on ESBL status alone. These findings emphasize that ESBL production, a routinely detectable feature in clinical microbiology laboratories, may serve as a valuable early warning indicator of broader antimicrobial resistance in *Salmonella* isolates. This could support empirical treatment optimization and infection control measures in low-resource settings.

## Discussion

### Sociodemographic characteristics of participants

A total of 265 patients were enrolled in the study, spanning a wide age range of 1 to 74 years. The age distribution revealed that adults aged 25–44 years constituted the largest demographic subgroup (30.2%), followed closely by children aged 1–14 years (27.9%). The cohort also included youths and young adults (15–24 years), representing 18.1%, and middle-aged adults (45–64 years) at 17.0%. Notably, elderly participants (≥65 years) accounted for 6.8% of the study population (Fig. 1a). This distribution reflects a relatively young population structure consistent with national demographic trends in Nigeria [58]. In terms of sex distribution, there was a slight male predominance, with 143 males (54.0%) and 122 females (46.0%) enrolled in the study (Fig. 1b). This male-to-female ratio aligns with other hospital-based studies in sub-Saharan Africa, where care-seeking behaviors, access disparities, and sociocultural factors often influence sex distribution in clinical research populations [59–61]. The balanced representation across both age and sex enhances the generalizability of the study findings and supports subgroup analyses on resistance patterns, which are known to vary with host demographic factors [61, 62].

### Objective 1: prevalence of MDR and ESBL among clinical *salmonella* isolates

#### Prevalence and serogroup distribution

This study documented a 24.5% prevalence of Salmonella spp. among patients presenting with gastrointestinal symptoms at a tertiary hospital in Nigeria. Notably, no Salmonella isolates were recovered from blood cultures—a statistically significant difference when compared to stool isolate recovery (*p* < 0.02). The absence of bloodstream isolates likely reflects a confluence of factors, including pre-hospital antibiotic exposure, low bacterial load in mild infections, and the limited sensitivity of conventional culture-based methods, particularly in endemic and resource-constrained settings [63–67]. Similar diagnostic trends have been observed across sub-Saharan Africa, including Ghana, Kenya, and Malawi, where delayed presentation and empirical antibiotic use reduce culture positivity [32, 68].

The serogrouping results, derived from O-antigen agglutination due to laboratory constraints, identified Group D1 (69%)—typically associated with *Salmonella enterica* serovar Typhi—as the predominant serogroup, followed by Groups B (19%) and C1 (12%). This distribution echoes findings from other Nigerian and West African studies [69, 70], highlighting the co-circulation of typhoidal and non-typhoidal Salmonella (NTS) pathovars. The coexistence of these strains reinforces the need for integrated diagnostics and surveillance, capable of differentiating between pathovars due to their distinct clinical trajectories and treatment approaches.

#### Burden of MDR and ESBL production

The burden of antimicrobial resistance was substantial. Among the 65 confirmed isolates, 43.1% met the definition of multidrug resistance (MDR)—resistance to ≥ 3 antibiotic classes—while 27.7% were confirmed to be ESBL producers via double-disk synergy testing. These figures are consistent with recent reports from Nigerian tertiary hospitals [21–23] and are reflective of escalating global trends in resistance among *Salmonella* species, particularly in low- and middle-income countries (LMICs) where cephalosporins and fluoroquinolones remain the mainstay of empirical therapy [71–73].

Of particular concern is the synergistic relationship between ESBL production and MDR, with 94.4% of ESBL-producing isolates exhibiting MDR, compared to 78.1% of non-ESBL isolates. This pattern supports the hypothesis of genetic co-selection of resistance determinants—whereby genes encoding ESBLs (e.g., *blaTEM*) co-localize with other resistance markers (e.g., *tetA*, *sul1*) on integron-bearing plasmids [68, 74–76]. Such linkage enhances the likelihood of horizontal gene transfer, particularly in settings with high antibiotic selection pressure.

#### Clinical and public health implications

The Multiple Antibiotic Resistance Index (MARI) was significantly higher among ESBL producers (0.62 vs. 0.48; *p* < 0.05), suggesting greater antibiotic exposure—either from nosocomial sources or inappropriate over-the-counter antibiotic use. Clinically, these findings complicate treatment algorithms for gastroenteritis and enteric fever in Nigeria, where diagnostic delay, limited AST access, and empirical therapy dominate care pathways [77].

From a public health perspective, the high proportion of MDR and ESBL-producing isolates in community-acquired samples raises red flags for resistance dissemination beyond the healthcare system. This supports calls for:

- Strengthening routine phenotypic resistance screening in stool samples.
- Expanding molecular surveillance to map resistance genes.
- Enhancing antibiotic stewardship at the community and facility levels.

### Objective 2: distribution of resistance genes among MDR and ESBL-positive isolates

#### Key findings and interpretations

Molecular profiling of antimicrobial resistance genes revealed a striking dominance of *blaTEM* (77.8%), followed by *tetA* (72.2%) and sul1 (61.1%) among *Salmonella* isolates co-expressing MDR and ESBL phenotypes. These three genes accounted for the majority of detected resistance determinants and were often co-expressed in the same isolates, pointing to a co-localization on mobile genetic elements such as class 1 integrons or resistance plasmids. The *blaTEM* gene, in particular, has emerged as the most prevalent extended-spectrum β-lactamase (ESBL) genotype in multiple settings across sub-Saharan Africa and Latin America [75, 76, 78, 79], and its dominance in our cohort underscores a continuing regional and global trend.

Notably, *blaSHV* was found in only 22.2% of ESBL-producing isolates, reaffirming its lower prevalence in *Salmonella* relative to other Enterobacteriaceae, such as *Klebsiella pneumoniae* or *Escherichia coli* [80–82]. The co-detection of *blaTEM* with *tetA* and *sul1* further strengthens the hypothesis of integron-mediated resistance clustering, where co-selection pressures may arise from the use of non–β-lactam antibiotics like tetracyclines and sulfonamides, sustaining β-lactam resistance even when direct selection is absent [83].

#### Comparative genomics and qnr gene absence

A particularly relevant finding was the complete absence of *qnrA* and *qnrB* genes, which encode plasmid-mediated quinolone resistance (PMQR). This stands in contrast to studies from South and Southeast Asia—especially India and Pakistan—where qnr gene carriage among ESBL-producing *Salmonella* is frequent and often correlates with fluoroquinolone resistance [84, 85]. The absence of qnr genes in our study may indicate that fluoroquinolone resistance in this population is driven predominantly by chromosomal mechanisms, such as point mutations in *gyrA* or *parC*, a possibility also supported by regional findings from West Africa [28].

This divergence from Asian genomic resistance patterns is significant, suggesting that global resistance prediction tools may underperform unless tailored to regional genotypic landscapes. It further implies that surveillance in Nigeria and similar LMICs should prioritize detection of chromosomal mutations and non-qnr quinolone resistance pathways.

#### Diagnostic and surveillance implications

These results have important implications for diagnostic microbiology and molecular surveillance. Given the high co-occurrence of *blaTEM* with *tetA* and *sul1*, the development of multiplex PCR panels or point-of-care molecular diagnostics targeting these gene clusters could provide rapid, cost-effective triage in resource-constrained laboratories. Such tools may serve as early warning systems for MDR risk, especially in settings without access to whole-genome sequencing (WGS) or automated AST platforms [86, 87].

Further, integrating genotypic surveillance with routine AST workflows may improve prediction of phenotypic resistance outcomes, optimize empirical therapy, and enhance detection of emerging resistance trends. As antimicrobial resistance continues to evolve, the combination of resistance gene profiling with machine learning–based classification models, as explored in Objectives 4 and 5, holds substantial promise for improving outbreak response, risk stratification, and stewardship interventions.

### Objective 3: association between Resistance genes and phenotypic resistance

#### Key findings and clinical correlation

The presence of ESBL production among *Salmonella* isolates in this study was strongly associated with phenotypic resistance to multiple antibiotic classes. Notably, 97.6% of ESBL-producing isolates were resistant to cefotaxime (CTX)—a third-generation cephalosporin—confirming the functional expression of β-lactamase enzymes, particularly those of the TEM type. In addition, high rates of co-resistance were observed for amoxicillin-clavulanic acid (AMC: 97.6%), sulfamethoxazole-trimethoprim (SXT: 83.3%), ciprofloxacin (CIP: 69.1%), and nalidixic acid (NA: 97.6%), underscoring the multidrug resistance (MDR) complexity harbored by ESBL-producing strains.

This phenotypic constellation mirrors resistance patterns documented in studies from Nigeria, Ghana, and India, where co-resistance to fluoroquinolones, sulfonamides, and aminopenicillins among ESBL-producing *Salmonella* isolates has been increasingly reported [41, 88, 89].

#### Functional genotype–phenotype concordance

The strength of the genotype–phenotype relationship was quantitatively affirmed by multivariable logistic regression analysis. ESBL production independently predicted cefotaxime resistance (aOR = 22.7, *p* < 0.01) and ciprofloxacin resistance (aOR = 3.3, *p* = 0.04), further validating the diagnostic value of phenotypic β-lactamase screening.

Although the MDR phenotype approached significance (aOR = 4.5, *p* = 0.05), this trend highlights the potential of ESBL positivity as a marker for broader antimicrobial resistance, even beyond β-lactam agents. Importantly, these data align with the One Health framework, wherein antimicrobial pressure across human, animal, and environmental domains facilitates the co-transmission of β-lactam and non–β-lactam resistance traits [90, 91].

#### Functional insights:

Triple Gene Co-Expression and Resistance Islands

A central molecular finding in this study was the frequent co-occurrence of *blaTEM, tetA*, and *sul1* genes within the same ESBL-producing isolates. This genetic clustering is likely mediated by class 1 integrons and multidrug resistance (MDR) plasmids, facilitating horizontal gene transfer and accelerating dissemination [83, 90].

Importantly, this triple gene signature tracked closely with phenotypic resistance to cephalosporins, tetracyclines, and sulfonamides, respectively. Similar triads have been documented in Latin America and West Africa, where co-expression confers an adaptive advantage under broad-spectrum antimicrobial pressure [75–77]. This observation supports integrative molecular surveillance frameworks capable of capturing resistance evolution via mobile genetic platforms.

#### Therapeutic implications and stewardship recommendations

These data reinforce the urgency of refining empirical treatment protocols, particularly in low- and middle-income countries (LMICs) where third-generation cephalosporins and fluoroquinolones remain frontline agents. The observed resistance convergence severely limits therapeutic options and shifts reliance toward carbapenems or colistin—drugs that are costly, less accessible, and should be reserved for last-line use [92–95].

In the absence of real-time molecular testing, phenotypic ESBL detection may serve as a pragmatic proxy for anticipating broader MDR risk. This is particularly relevant in endemic regions where laboratory infrastructure is limited, and treatment delays exacerbate mortality.

### Objective 4: predictive utility of resistance markers for MDR and ESBL classification

#### 4a. Statistical predictors: logistic regression and cart models

The multivariate logistic regression analysis confirmed that cefotaxime (CTX) resistance, along with the presence of *blaTEM* and *tetA*, were statistically significant predictors of both MDR and ESBL phenotypes. While CTX resistance emerged as the strongest independent predictor of MDR status, *blaTEM* and *tetA* remained significant in identifying ESBL-producing strains. This aligns with previous studies across South Asia and sub-Saharan Africa that link third-generation cephalosporin resistance with high-risk mobile genetic elements carrying multiple resistance traits [84, 85].

To visualize classification logic, CART (Classification and Regression Tree) models were developed for both MDR and ESBL phenotypes (see Figs. 4 and 5). These trees placed CTX resistance at the root node in both cases, with sequential branching based on ciprofloxacin resistance and *tetA* gene presence, highlighting how resistance pathways converge around β-lactam pressure.

By combining both genotypic and phenotypic predictors, these models offered intuitive decision frameworks, especially valuable in resource-limited settings where access to sequencing or ensemble machine learning is often constrained [96, 97].

#### 4b. Machine learning performance: random forest Classifier

To explore non-linear patterns beyond traditional regression and decision trees, a Random Forest model was trained to classify MDR and ESBL phenotypes. As summarized in Figure 7 and Table 11, the ensemble model outperformed logistic regression and CART, demonstrating:

- **MDR Classification**: Accuracy = 97.6%, AUC = 0.95
- **ESBL Classification**: AUC = 0.99, Specificity = 100%

These results affirm the capability of machine learning models to detect complex interactions between resistance genes and phenotypic traits that classical methods may overlook. The strong performance of *blaTEM* and CTX resistance as individual predictors is captured in Fig. 8, which plots comparative ROC curves.

To avoid redundancy, detailed Area Under the Curve (AUC) values are not repeated throughout the text; comparative performance of individual predictors is instead summarized visually in the ROC overview (Fig. 8). The relatively low AUC for ciprofloxacin resistance (~0.55) suggests that fluoroquinolone resistance may not be central to MDR determination in this cohort, in contrast with findings from Asia [29, 83, 98, 99].

#### 4c. Interpretability via SHAP framework

A notable advancement in this study was the use of SHAP (Shapley Additive Explanations) to explain Random Forest predictions at both global and individual levels.

- The SHAP summary plot (Figure 10a) identified CTX resistance, *blaTEM*, and *tetA* as the top global contributors to MDR classification—mirroring findings from regression and CART models.
- An individual-level SHAP waterfall plot (Figure 10b) demonstrates how these predictors shift model probability from baseline to MDR-positive, offering interpretability critical to clinician trust and policy integration [100].

By providing both statistical strength and model transparency, SHAP bridges the gap between computational sophistication and bedside application, particularly in microbiology labs within LMICs where capacity for full-scale genomic interpretation remains limited [101, 102].

#### Surveillance and clinical utility

The combination of classical and machine learning methods in this objective illustrates a scalable framework for predictive diagnostics. In settings with delayed phenotypic AST turnaround times, the presence of CTX resistance and *blaTEM* may serve as early flags for MDR risk, guiding empirical therapy and infection control protocols.

Furthermore, by integrating SHAP-enhanced Random Forest models into laboratory information systems, real-time flagging of high-risk isolates becomes possible, enabling more timely antimicrobial stewardship interventions [103–105].

### Objective 5: predictive value of ESBL status for multidrug resistance (MDR)

#### Key findings and diagnostic implication

This study identified ESBL phenotype as a statistically significant predictor of multidrug resistance (MDR) in clinical *Salmonella* isolates. Univariate logistic regression demonstrated that ESBL-positive isolates were 4.6 times more likely to exhibit MDR traits than ESBL-negative counterparts (aOR = 4.6; 95% CI: 1.5–14.3; *p* < 0.01). This predictive association is further validated by the Receiver Operating Characteristic (ROC) analysis (Fig. 6), which produced an AUC of 0.79, indicating a moderate-to-strong discriminative ability.

These results are consistent with poor resource countries reports such from Ghana, India, and Colombia, where ESBL-producing Enterobacteriaceae—especially those expressing *blaTEM* and *CTX-M* genes—frequently exhibit co-resistance to multiple antimicrobial classes [76, 88, 89, 106]. The strength of this association highlights the diagnostic utility of ESBL screening as a surrogate marker for MDR risk in resource-limited settings where comprehensive susceptibility profiling may be delayed or unavailable.

#### Genomic basis: co-selection on Mobile elements

As established earlier (see Objective 2 and 3), the observed ESBL–MDR correlation is mechanistically underpinned by the co-occurrence of plasmid-borne resistance genes, particularly *blaTEM*, *tetA*, and *sul1*. These genes, often harbored within class 1 integrons or conjugative plasmids, facilitate horizontal gene transfer and co-selection—wherein exposure to one antibiotic class can select for resistance traits across others [83, 90].

This insight reinforces the need for multiplex resistance gene surveillance rather than single-gene diagnostics, as co-selection and gene linkage complicate resistance control strategies.

#### Clinical utility: triage and stewardship

In clinical contexts, particularly in low- and middle-income countries (LMICs) where AST delays or stockouts are common, phenotypic ESBL detection methods (e.g., double-disc synergy tests) offer a practical proxy for identifying high-risk patients. As established earlier, ESBL phenotype offers a reliable proxy for MDR risk, and this can inform early empirical therapy, patient isolation, and escalation of care.

Evidence from Kenyan and Bangladeshi hospitals suggests that incorporating ESBL status into decision trees reduces time to effective therapy and improves patient outcomes [84, 107, 108].

#### Public health and one health implications

From a One Health perspective, the convergence of ESBL production and MDR traits in *Salmonella* isolated from community-acquired fecal samples suggests broader ecological transmission dynamics. The findings mirror trends from community settings in Ethiopia and South Asia, where indiscriminate use of antimicrobials in livestock, unregulated pharmacy sales, and poor sanitation amplify resistance dissemination [109, 110].

Routine ESBL screening, therefore, plays a dual role: aiding clinical triage and serving as a sentinel surveillance tool in community and hospital settings. These findings advocate for the institutionalization of ESBL detection within national AMR surveillance frameworks, alongside molecular typing and genomic tracing of resistance determinants.

A key limitation of this objective is the absence of whole-genome sequencing (WGS), which restricts definitive linkage between ESBL production, MDR phenotype, and specific plasmid or clonal backgrounds. Future work should integrate WGS to map resistance islands, track clonal spread, and better understand gene flow between human, animal, and environmental reservoirs [40, 111].

## Conclusion

This study provides crucial insights into the molecular and phenotypic underpinnings of antimicrobial resistance among *Salmonella* isolates from a tertiary hospital in Nigeria, highlighting the convergence of extended-spectrum β-lactamase (ESBL) production and multidrug resistance (MDR) within a high-burden setting. The predominance of *blaTEM*, *tetA*, and *sul1* genes, often co-occurring within the same isolates, points to the likely co-location of resistance determinants on integron-bearing plasmids—underscoring the role of horizontal gene transfer in amplifying resistance within clinical and environmental reservoirs.

The study demonstrates that phenotypic markers such as cefotaxime resistance, in conjunction with genotypic markers like *blaTEM* and *tetA*, serve as robust predictors of MDR and ESBL phenotypes. This predictive utility was reinforced through logistic regression, CART analysis, and machine learning models, with Random Forest classifiers achieving high sensitivity and specificity. Importantly, the integration of SHAP-based interpretability enhanced transparency in model predictions and facilitated clinical relevance.

Furthermore, the study establishes that ESBL status is a reliable surrogate marker for MDR, offering a practical alternative in settings where advanced molecular diagnostics are unavailable. These findings have both diagnostic and surveillance implications—supporting the development of multiplex panels, early-warning systems, and stewardship strategies tailored to low-resource environments.

In sum, the work contributes to the growing body of literature emphasizing the need for integrated diagnostic platforms that leverage both genotypic and phenotypic data for real-time resistance prediction. It underscores the urgency of strengthening laboratory capacity, implementing routine ESBL screening, and expanding surveillance infrastructure as part of Nigeria’s national action plan on antimicrobial resistance (AMR). Future studies should incorporate whole-genome sequencing to elucidate clonal relationships, resistance islands, and transmission pathways, thereby advancing precision epidemiology and One Health interventions.

### General discussion

Synthesis of Key Findings

Across five defined objectives, this study provides a comprehensive resistance profile of clinical *Salmonella* isolates:

- **Objective 1** established a 43.1% prevalence of MDR and 27.7% of ESBL production, with significantly higher MARI scores in ESBL-positive isolates, indicating greater cumulative antibiotic exposure.
- **Objective 2** identified *blaTEM*, *tetA*, and *sul1* as the dominant resistance genes, frequently co-occurring in MDR and ESBL isolates—suggesting horizontal gene transfer and possible integron-mediated mobility.
- **Objective 3** showed that ESBL producers exhibited significantly elevated resistance to cephalosporins, fluoroquinolones, and sulfonamides, confirming phenotypic expression of genotypic resistance mechanisms.
- **Objective 4** highlighted that CTX resistance, *blaTEM*, and *tetA* were consistently top predictors of MDR and ESBL, validated across logistic regression, CART, and Random Forest models. SHAP analysis further enhanced interpretability by quantifying the contribution of each variable to resistance predictions.
- **Objective 5** confirmed that ESBL production was strongly associated with MDR (aOR = 4.6), with an AUC of 0.79—positioning ESBL phenotype as a useful surveillance proxy in the absence of full molecular diagnostics.

### Clinical and public health implications

From a clinical standpoint, resistance to third-generation cephalosporins and fluoroquinolones compromises first-line treatment options, narrowing therapeutic windows to last-resort agents like carbapenems and polymyxins. This trajectory is unsustainable and reinforces the urgent need for antimicrobial stewardship, especially in high-burden, low-resource settings. The identification of reliable, low-cost predictors of resistance (e.g., ESBL phenotype, *blaTEM*) offers immediate translational value in empirical treatment decision-making.

### Surveillance and policy relevance

The integration of machine learning into diagnostic workflows—particularly Random Forest models enhanced by SHAP-based interpretation—holds potential for automated resistance forecasting, prioritizing high-risk isolates, and triggering infection control protocols. Moreover, the documented gene co-occurrence patterns should inform molecular surveillance frameworks and antibiotic regulation policies at both hospital and national levels.

### Study limitations and future directions

The inability to perform whole genome sequencing limited insights into clonal relationships and full resistome mapping. Additionally, the study’s cross-sectional design constrains temporal inferences. Future work should incorporate longitudinal sampling, WGS, and resistance plasmid profiling to unravel transmission dynamics and support region-specific containment strategies.

### Justification for dataset size

Although the dataset used for machine learning analysis was relatively limited in size, its use is methodologically justified. The study prioritized high-quality, well-characterized *Salmonella* isolates with complete phenotypic and genotypic profiles to ensure data integrity and analytical reliability. Given the resource constraints inherent in molecular surveillance studies in low- and middle-income settings, the emphasis on data quality over quantity was deliberate. Furthermore, the selected algorithms—Logistic Regression, CART, Random Forest, and GAM—are robust to moderate sample sizes when appropriate cross-validation and feature selection procedures are applied. The modeling approach was intended primarily for exploratory identification of key predictors rather than large-scale prediction, thus providing a valid and informative foundation for subsequent studies with larger, multicenter datasets.

## Data Availability

All data collected and analysed during this study are available in this manuscript and on request from the corresponding author.

## Abbreviations

AE- FUTHA: Alex Ekwueme Federal University Teaching Hospital
AMR: Antimicrobial resistance
ANOVA: Analysis of variance
AUC: Area under the curve
CART: Classification and Regression Tree
CLSI: Clinical and Laboratory Standards Institute
DDST: Double Disc Synergy Test
ESBL: Extended-spectrum beta-lactamase
GAM: Generalized Additive Models
HGT: Horizontal gene transfer
LMICs: Low- and Middle- income Countries
MARI: Multiple antibiotic resistance index
MDR: Multidrug Resistance
NTC: No-template control
NTS: Non-typhoidal *Salmonella*
PCR: Polymerase Chain Reaction
PMQR: Plasmid-Mediated Quinolone Resistance
ROC: Receiver Operating Characteristic
SHAP: Shapley Additive Explanations
SPPS: Statistical Package for Social Sciences
TSI: Triple sugar iron
WGS: Whole-genome sequencing
WHO: World Health Organization
XLD: Xylose-Lysine-Deoxycholate
